# Hepatoprotective Effect of Cereal Vinegar Sediment in Acute Liver Injury Mice and Its Influence on Gut Microbiota

**DOI:** 10.3389/fnut.2021.798273

**Published:** 2021-12-24

**Authors:** Qijie Guan, Tingting Gong, Zhen-Ming Lu, Yan Geng, Wenhui Duan, Yi-Lin Ren, Xiao-Juan Zhang, Li-Juan Chai, Jin-Song Shi, Zheng-Hong Xu

**Affiliations:** ^1^Key Laboratory of Industrial Biotechnology, Ministry of Education, Jiangnan University, Wuxi, China; ^2^National Engineering Laboratory for Cereal Fermentation Technology, Jiangnan University, Wuxi, China; ^3^School of Pharmaceutical Science, Jiangnan University, Wuxi, China; ^4^Jiangsu Engineering Research Center for Bioactive Products Processing Technology, Jiangnan University, Wuxi, China; ^5^Department of Gastroenterology, Affiliated Hospital of Jiangnan University, Wuxi, China

**Keywords:** cereal vinegar sediment, composition analysis, liver injury prevention, gut microbiota, CCl_4_ induced liver injury

## Abstract

Cereal vinegar sediment (CVS) is a natural precipitate formed during the aging process of traditional grain vinegar. It has been used as Chinese traditional medicine, while its composition and function are reported minimally. In this study, we measured CVS in terms of saccharide, protein, fat and water content, and polyphenol and flavonoid content. Furthermore, we determined the amino acids, organic acids, and other soluble metabolites in CVS using reverse-phase high-performance liquid chromatography (RP-HPLC), HPLC, and liquid chromatography with tandem mass spectrometry (LC-MS/MS) platforms. The hepatoprotective effect of CVS was evaluated in acute CCl_4_-induced liver injury mice. Administration of CVS for 7 days prior to the CCl_4_ treatment can significantly decrease liver alanine aminotransferase (ALT) and aspartate aminotransferase (AST) levels and reactive oxygen species (ROS) levels, compared with those in the hepatic injury model group. The gut microbiota was changed by CCl_4_ administration and was partly shifted by the pretreatment of CVS, particularly the Muribaculaceae family, which was increased in CVS-treated groups compared with that in the CCl_4_ administration group. Moreover, the abundances of *Alistipes* genus and Muribaculaceae family were correlated with the liver ALT, AST, and malondialdehyde (MDA) levels. Our results illustrated the composition of CVS and its hepatoprotective effect in mice, suggested that CVS could be developed as functional food to prevent acute liver injury.

## Introduction

Cereal vinegar sediment (CVS) is a natural precipitate during the aging process of traditional solid-state fermented vinegar with grain as raw material. Vinegar sediment has reported pharmacological activities in several human diseases, including hyperlipidemia (1), hyperglycemia (2), allergic diseases (3), dermatitis (4), senile dementia (5), and osteoporosis (6). Vinegar sediment also has several effects on the intestinal tract. Fukuyama et al. (7) found that Japanese black vinegar *Kurozu Moromi* paste inhibited the development of colon cancer in mice, but vinegar itself could not inhibit the growth of colon cancer. Shizuma et al. (8) found that vinegar had anti-colitis and anti-oxidation effects in dextran sulfate sodium-induced mice models and speculated that its amino acids and oligopeptides, and other organic substances, might be biologically active. It was also found that *Kurozu Moromi* could inhibit the growth of hepatoma cells and prolong the survival time of hepatoma mice (9). However, the research on its material basis and mechanism of liver protection is still insufficient.

Gut–liver communications play important role in liver diseases (10). The growing evidence indicates that the pathogenetic role of microbe-derived metabolites, such as trimethylamine, secondary bile acids, short-chain fatty acids, and ethanol, in the pathogenesis of the non-alcoholic fatty liver disease (NAFLD) (11), modulation of the gut microbiota may represent a new way to treat or prevent NAFLD (12). Two kinds of traditional vinegar in China, Shanxi aged vinegar and Hengshun aromatic vinegar, have hepatoprotective effects through antioxidants and regulating the blood lipid levels and inflammatory response levels (13, 14). Shanxi aged vinegar extract could modulate gut microbiota, improve intestinal homeostasis, and can be used as a novel gut microbiota manipulator against alcohol-induced liver damage (15). Moreover, monascus vinegar could reduce lipids, intestinal *Lactobacillus, Roseburia*, and *Lachnoclostridium*, which had a positive correlation with antioxidative parameters and a negative correlation with lipid metabolism (16). Waste vinegar residue, which was similar to CVS, was able to help modify intestinal pH value and affect the lower gut microflora (17).

However, studies aiming to illustrate the bioactive functions of vinegar and vinegar by-products are still limited. In the present study, we measured the chemical compositions in CVS and explored the hepatoprotective effect of CVS on acute liver injury in mice induced by CCl_4_. These findings may illuminate the potential mechanism of CVS on intestinal microbiota and acute liver injury.

## Materials and Methods

### Preparation of CVS and Chemical Analysis

The vinegar was made with a traditional solid-state fermentation method, based on Shanxi aged vinegar fermentation method (18), using sorghum as raw material in Zhangjiajie, Hunan Province, China. The total acidity of vinegar products is 6% (w/v), including 2.1% (w/v) non-volatile acid. The vinegar was naturally aged in pottery jars for 5 years. After aging, aged vinegar and its natural sediment were mixed and centrifuged with 7,500 × *g* for 20 min. The sediment was collected as CVS after the centrifugation.

Because of the lack of chemical composition in CVS, the authors applied multiple analyses to illustrate the construction of CVS. The measurements include the water content (19), the crude protein and fat content (20), total saccharide and polysaccharide content (21), total polyphenol content (22), and total flavonoid content (23).

### Reverse-Phase High-Performance Liquid Chromatography Analysis

Amino acids were determined by using reverse-phase high-performance liquid chromatography (RP-HPLC) via pre-column derivatization with O-phthalaldehyde (OPA) and 9-fluorenylmethyl chloroformate (FMOC-Cl). The 17 amino acid standards for quantification included glutamic acid, proline, aspartic acid, glycine, alanine, arginine, histidine, serine, tyrosine, cysteine, valine, leucine, isoleucine, phenylalanine, threonine, lysine, and methionine.

An Agilent 1100 HPLC (NYSE: A; Agilent Technologies Inc., Palo Alto, CA, USA) and an Agilent Hypersil ODS column (5 μm, 4.0 × 250 mm) were applied for RP-HPLC analysis. A 27.6 mM sodium acetate–triethylamine–tetrahydrofuran (500:0.11:2.5, v/v/v, pH = 7.2) was used as solvent A, and an 80.9 mM sodium acetate–ethanol–acetonitrile (1:2:2, v/v/v, pH = 7.2) was used as solvent B for mobile phases at a flow rate of 1 ml/min. The elution gradient employed was as follows: 0–17 min, 8–50% B; 17–20 min, 50–100% B; 20–24 min, 100–0% B. Amino acids were detected by ultraviolet (UV) detector at 338 nm, whereas proline was detected at 262 nm.

### HPLC Analysis

Ten milliliter distilled water was added to 1 g CVS, shaken fully, and centrifuged. About 5 ml supernatant, 2 ml 0.25 M potassium ferrocyanide, and 2 ml 30% (w/v) zinc sulfate were mixed and then centrifuged. The organic acids in the supernatant were analyzed by using HPLC with Waters Atlantis T3 column (5 μm, 4.6 × 250 mm; Waters Corp., Milford, MA, USA) and quantified by external standard methods. The mobile phase was 20 mM NaH_2_PO_4_ (pH = 2.7), flowing at a speed of 0.7 ml/min. Injection volume was 10 μl. It was detected by a UV detector at 210 nm. Seven kinds of organic acid standards were used for quantification included oxalic acid, tartaric acid, pyruvic acid, lactic acid, acetic acid, citric acid, and succinic acid.

### LC-MS/MS Analysis

For each CVS replicate, 600 μl 2-chlorophenylalanine in 80% methanol was added to 100 mg CVS sample, vortexed for 30 s. After the vortex, 100 mg glass beads were added into the sample tube, the sample was ground in a tissue grinder for 90 s at 60 Hz. After ultrasonication, the sample was centrifuged at 8,000 g at 4°C for 10 min. About 300 μl supernatant was filtered through a 0.22-μm membrane, and the filtrate was collected for further analysis.

A Vanquish liquid chromatography system connected to a Q Exactive HF-X mass spectrometer (Thermo Fisher Scientific, Bremem, Germany) was used for untargeted metabolomics analysis. The metabolites were resuspended in a loading solvent and loaded onto an ACQUITY UPLC^®^ HSS T3 (150 × 2.1 mm, 1.8 μm, Waters Corp., Milford, MA, USA) column. Gradient elution of analytes was carried out with 0.1% formic acid in water (A1) and 0.1% formic acid in acetonitrile (B1) or 5 mM ammonium formate in water (A2) and acetonitrile (B2) at a flow rate of 0.25 ml/min. Injection of 2 μl of each sample was done after equilibration. An increasing linear gradient of solvent B (v/v) was used as follows: 0–1 min, 2% B2/B1; 1–9 min, 2–50% B2/B1; 9–12 min, 50–98% B2/B1; 12–13.5 min, 98% B2/B1; 13.5–14 min, 98–2% B2/B1; and 14–20 min, 2% B1-positive model (14–17 min, 2% B2-negative model). The analyzer was scanned over a mass range of *m*/*z* 81–1,000 for the full scan at a mass resolution of 60,000. Data-dependent acquisition (DDA) MS/MS experiments were performed with higher-energy collisional dissociation (HCD) scan. The MS/MS data were then searched with Human Metabolome Database (HMDB) (http://www.hmdb.ca), METLIN (http://metlin.scripps.edu), Massbank (http://www.massbank.jp), LipidMaps (http://www.lipidmaps.org), and mzClound (https://www.mzcloud.org) for metabolites identification.

### Animals and Treatments

**Figure 1:**
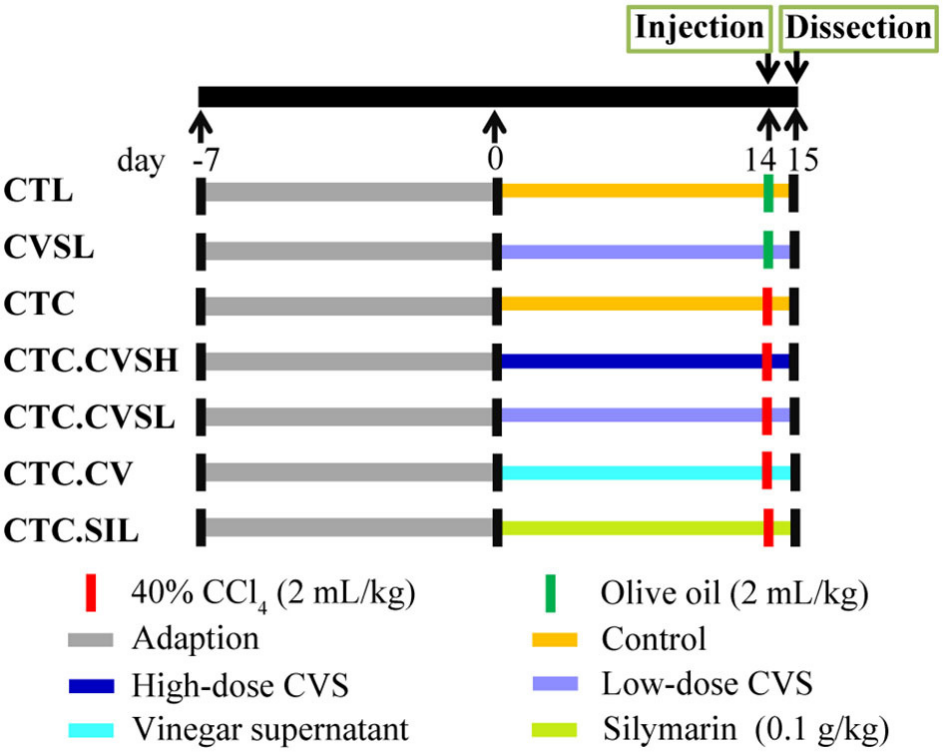
Animal experiences design of the study on cereal vinegar sediment (CVS) hepatoprotective effect.

Male Balb/c mice (6–8 weeks old) were purchased from Shanghai SLAC Laboratory Animal Co. Ltd., Shanghai, China. The animals were kept under specific-pathogen-free (SPF) conditions at 20–26°C and 40–70% relative humidity and acclimated for 1 week prior to use. Feed pellets and sterile water were provided for cafeteria feeding. All mice procedures and protocols were approved by the Institutional Animal Care and Use Committee of Jiangnan University, Wuxi, China [Approval No. JN. No 20170329-20170515 (24)]. Sixty mice were divided randomly into seven groups: normal control group (CTL), low-dose CVS treated group (CVSL), hepatic injury model group (CTC), high-dose CVS-treated hepatic injury group (CTC.CVSH), low-dose CVS-treated hepatic injury group (CTC.CVSL), vinegar-treated hepatic injury group (CTC.CV), and silymarin-treated hepatic injury group (CTC.SIL). Feeding was continued for 14 days. For each group, mice were equally divided into two cages at the beginning of the experiment, the concrete treatment was shown in Figure 1. About 1.4 g/kg dose of CVS was determined as high-dose CVS and 0.7 g/kg dose of CVS was determined as low-dose CVS according to the Kurozu dose (25) combined with our preliminary results (26). The CCl_4_-induced hepatic injury model was based on Taniguchi et al. (27); in our experiment, each hepatic injury model mouse was injected with 0.4 g/kg 40% CCl_4_ olive oil solution once (Figure 1). All solutions for intragastric administration used 0.5% CMC as solvent.

At the end of a week, feces in the cecum were collected after a 12-h fast. Mice were weighed and anesthetized via intraperitoneal injection of 1% sodium pentobarbital. Serum samples were collected from the eyeball to determine activities of serum aspartate aminotransferase (AST) and alanine aminotransferase (ALT), and the AST and ALT were measured using the corresponding kits (Nanjing Jiancheng Bioengineering Institute, Nanjing, China). All measurements were carried out with a microplate reader (Spectra max M5; Molecular Devices, San Jose, CA, USA).

The liver tissue and spleen were immediately excised, washed in normal saline, and weighed. The liver was cut into slices, some of which were kept in buffered formalin for histopathological observation, and the remaining were used to analyze hepatic malondialdehyde (MDA), superoxide dismutase (SOD), and catalase (CAT) levels.

### Determination of Liver Index and Spleen Index

The liver index of mice was calculated according to the following equation: tissue index = tissue weight (mg)/body weight (mg) × 100%.

The spleen index of mice was calculated according to the following formula: spleen index (mg/g) = spleen weight (mg)/animal body weight (mg) × 100%.

### Histological Examination

For the microscopic evaluation, the parts of the liver were fixed in 10% buffered formalin, embedded in paraffin, sectioned at 4 μm, and subsequently stained with hematoxylin-eosin (H&E). The magnifications were 100 times and 200 times with at least three areas of each tissue slice observed.

### Determination of Hepatic Antioxidant Index

Liver tissue was ground with a cross-paddle homogenizer, and 10% liver homogenate was prepared by adding normal saline. MDA levels along with SOD and CAT activities in the liver were measured using the corresponding kits (A001, A002, and A003; Nanjing Jiancheng Bioengineering Institute, Nanjing, China).

### 16S rRNA Sequencing and Analysis

Four fecal samples from each group were used for the DNA extraction using QIAamp DNA Stool Mini Kit (QIAGEN, Hilden, Germany) as directed in manual instructions. The V3 to V4 region of the bacterial 16S rRNA gene was amplified using the primers 338F (5′-barcode-ACTCCTACGGGAGGCAGCAG-3′) and 806R (5′-GGACTACHVGGGTWTCTAAT-3′). Barcodes were synthesized at the 5′ end of primer 338F to assign sequences for different samples. The PCR products were then checked by running a 2% agarose gel, purified using the GeneJET DNA Gel Extraction Kit (Thermo Fisher Scientific, Waltham, MA, USA), and quantified using Qubit (Thermo Fisher Scientific, Waltham, MA, USA). The purified amplifications were pooled and sequenced on Ion S5^TM^XL (Thermo Fisher Scientific, Waltham, MA, USA).

Cutadapt (28) was used for quality control and cutting of reads. The chimera sequences were detected by comparing them with a species annotation database (https://github.com/torognes/vsearch/) (29) and then removed. Uparse (30) was used for operational taxonomic unit (OTU) selection (at 97% similarity). A representative sequence was selected for each OTU and annotated with the taxonomic information in the SILVA132 SSUrRNA database (31) using Mothur. The confidence score threshold was set at 0.8, and sequences with a bootstrap value below 0.8 were assigned to an unclassified category. The resulting OTU table was further subsampled and followed by linear discriminant analysis effect size (LEfSe) analysis (32) and co-expression analysis based on the Pearson correlation coefficient (33).

### Statistical Analysis

Results are expressed as means ± SD. Statistical comparisons were calculated by python 3.7 (https://www.python.org/) using the Kruskal–Wallis test. Significance presents as: *p* < 0.001^***^, *p* < 0.01^**^, and *p* < 0.05^*^. There are also significant differences (*p* < 0.05) between data marked with different letters.

## Results

### Chemical Composition of CVS

**Figure 2:**
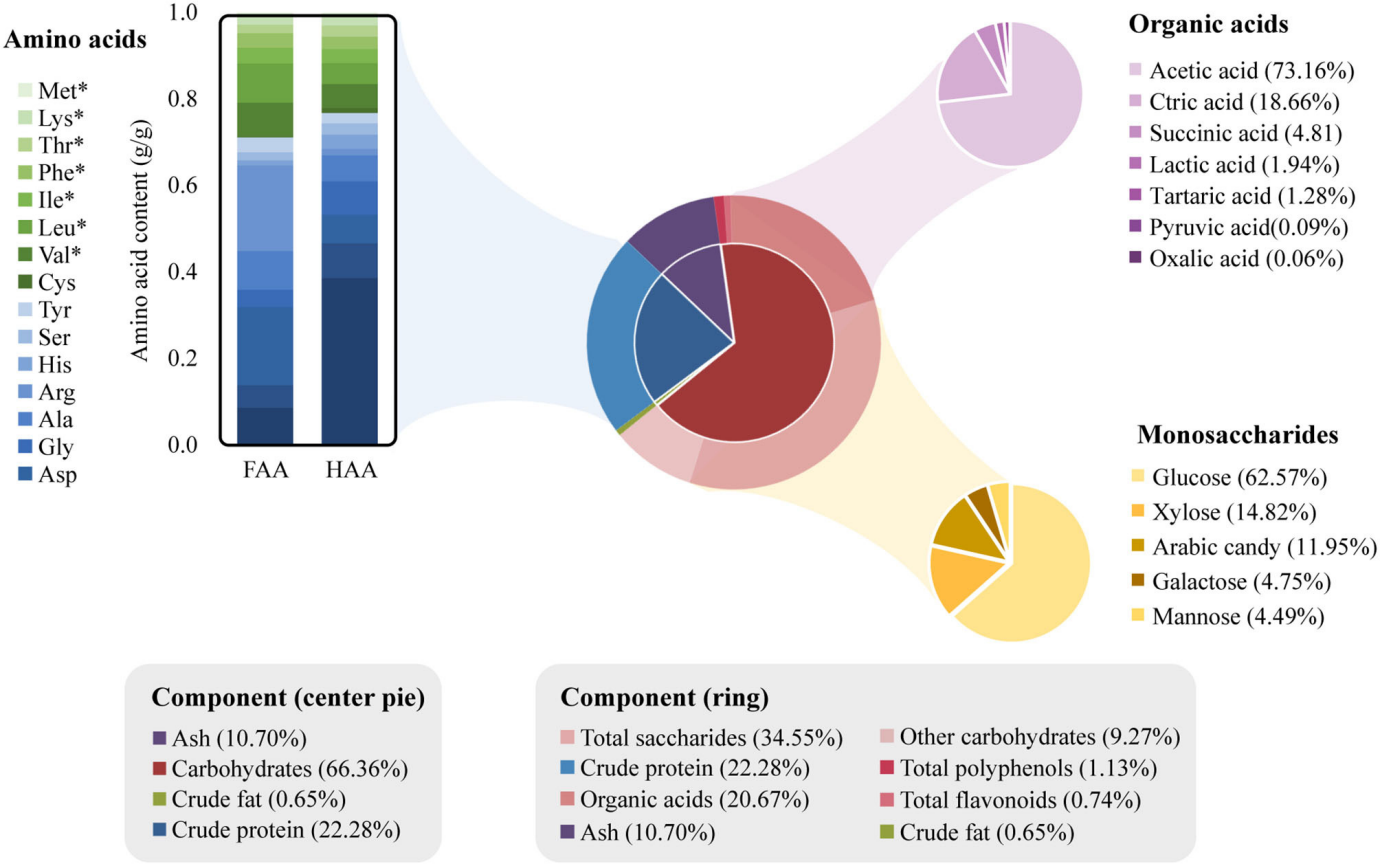
Chemical composition of cereal vinegar sediment (CVS). The four major components of CVS are shown in the center pie chart. The outer ring shows the specific components of crude protein and carbohydrates. The amino acids, organic acids, and monosaccharide composition of total saccharides are further shown in the bar, purple pie, and golden pie charts, respectively. The amino acids marked with asterisks (*) are essential amino acids.

The chemical analysis of CVS was summarized in Figure 2. The CVS contained 65% (w/w) water and 35% (w/w) dry matter. The contents of carbohydrates, crude protein, crude fat, and others in dry matter were 66.37% (w/w), 22.28% (w/w), 0.65% (w/w), and 10.70% (w/w). The CVS dry matter mainly consisted of carbohydrates and proteins. Compared with *Kurozu Moromi* (34), the CVS we used in this study had relatively a higher protein content.

The hydrolyzed amino acid (HAA) and free amino acid (FAA) contents inside CVS were measured and listed in Supplementary Table 1. About 100 g of CVS contained 4.68 g HAA and 0.43 g FAA. Glutamic acid, proline, glycine, aspartic acid, and alanine were the major non-essential amino acids. Valine content was highest in essential amino acids, followed by leucine, isoleucine, phenylalanine, threonine, lysine, and methionine. Total saccharide content (12.08% w/w) and polysaccharide content (0.39% w/w) in CVS was measured separately. The composition of saccharides was determined with hydrolyzation. Saccharides in CVS mainly consisted of glucosamine, xylose, arabinose, galactose, mannose, and glucose. Seven organic acids were determined and quantified in this study, including acetic acid (5.29% w/w), citric acid (1.35% w/w), succinic acid (0.35% w/w), lactic acid (0.14% w/w), tartaric acid (0.09% w/w), pyruvic acid (0.01% w/w), and oxalic acid (0.00% w/w). In addition, total polyphenol and total flavonoid content in CVS were derived from the calibration curve. The contents of polyphenols and flavonoids in CVS were 0.40 g GAE/100 g and 0.26 g RE/100 g, respectively.

From the liquid chromatography with tandem mass spectrometry (LC-MS/MS) analysis, we detected 3,175 metabolites from CVS (Supplementary Table 2), in which 379 metabolites had MS_2_ matching. As shown in Table 1, ten metabolites are having the largest peak areas in CVS including 5 acids, indicating the complexity of CVS.

**Table 1:** Top ten metabolites identified in CVS based on peak areas.

**Metabolite name**	**Formula**	**Retention Time**	**KEGG ID**	**Peak area (×10^9^)**
2-Hydroxypyridine	C_5_H_5_NO	102.5745	C02502	26.17 ± 4.38
6-Hydroxyhexanoic acid	C_6_H_12_O_3_	305.2525	C06103	20.95 ± 1.19
Succinic acid	C_4_H_6_O_4_	162.4425	C00042	12.21 ± 0.95
Erucic acid	C_22_H_42_O_2_	837.8375	C08316	8.32 ± 1.41
*p*-Coumaroylagmatine	C_14_H_20_N_4_O_2_	246.035	C04498	8.25 ± 2.96
Acetylcholine	C_7_H_16_NO_2_	102.1025	C01996	6.87 ± 0.69
Linatine	C_10_H_17_N_3_O_5_	234.234	C05939	6.81 ± 2.33
Pyridoxal	C_8_H_9_NO_3_	124.159	C00250	6.19 ± 1.67
Homocitrulline	C_7_H_15_N_3_O_3_	149.3205	C02427	6.18 ± 0.34
N-Acetyl-L-aspartic acid	C_6_H_9_NO_5_	145.8805	C01042	5.47 ± 0.33

### Effects of CVS on CCl_4_-Induced Liver Injury Mice

As shown in Supplementary Figure 1, after 7 days of high- and low-dose CVS, vinegar supernatant, and silymarin administrations, mice in the different groups grow similarly on body weights. After CCl_4_ and fasting treatment, the body weight decreased significantly as expected.

**Figure 3:**
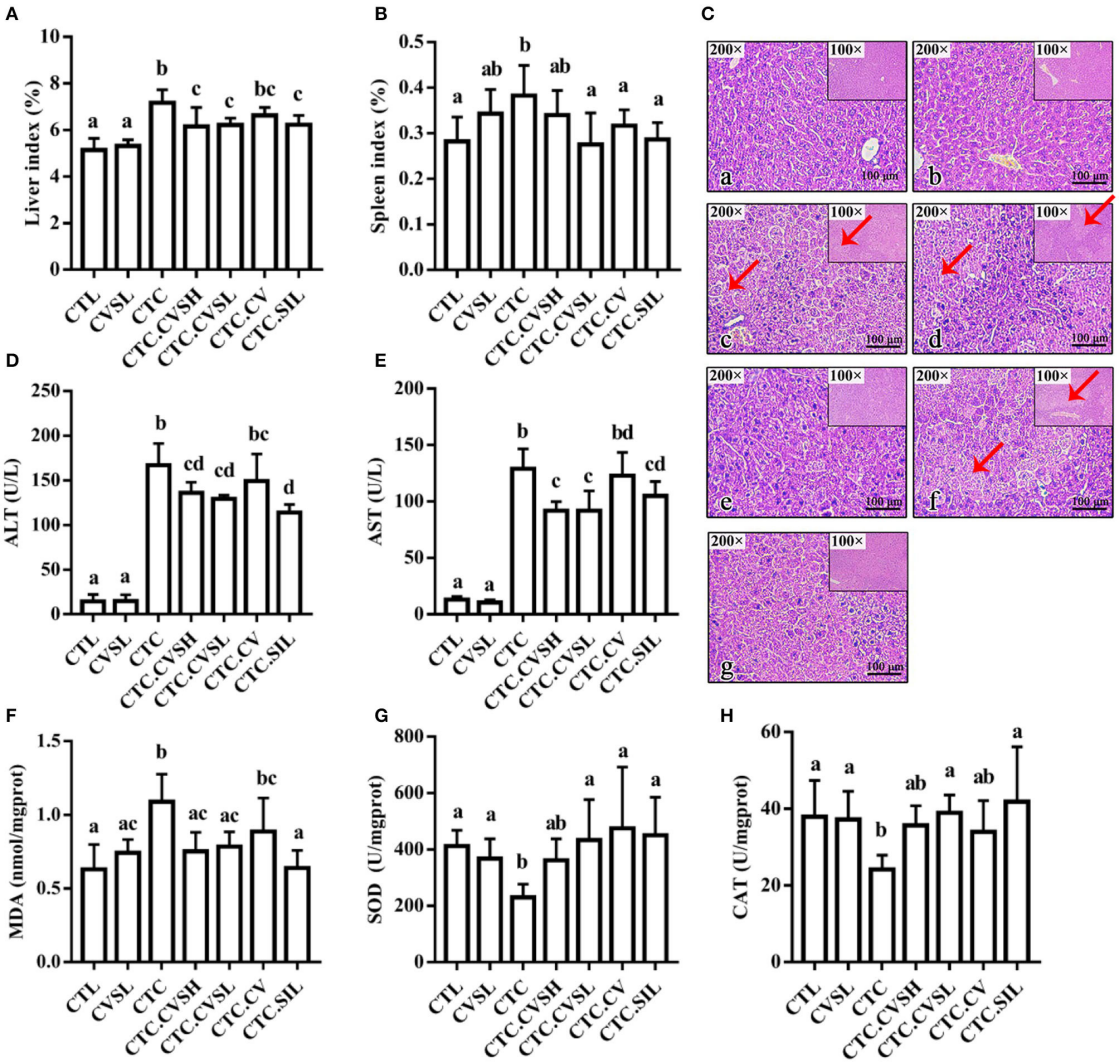
Liver-related indicators and the histology of the H&E stained liver under different treatments. **(A)** Liver index calculated in groups; **(B)** spleen index calculated in groups; **(C)** the histology of the H&E stained liver slices in the (a) CTL, (b) CVSL, (c) CTC, (d) CTC.CVSH, (e) CTC.CVSL, (f) CTC.CV, and (g) CTC.SIL groups. Scale bar is given in the figures. Red arrow points to hepatocyte without nuclei structure; **(D)** serum ALT levels measured in the groups; **(E)** serum AST levels measured in the groups; **(F)** hepatic MDA content levels measured in the groups; **(G)** SOD activity measured in the groups; **(H)** CAT activity measured in the groups. Different letters represent a significant difference between groups calculated with the Kruskal–Wallis one-way ANOVA, and error bars represent SD. AST, aspartate aminotransferase; CTL, normal control group; CVSL, low-dose CVS treated group; CTC, hepatic injury model group; CTC.CVSH, high-dose CVS-treated hepatic injury group; CTC.CVSL, low-dose CVS-treated hepatic injury group; CTC.CV, vinegar-treated hepatic injury group; CTC.SIL, silymarin-treated hepatic injury group; MDA, malondialdehyde; SOD, superoxide dismutase.

Free radicals generated during the CCl_4_ metabolism could cause liver damage. Thus, the CCl_4_ administration was expected to have a profound effect on the liver index, which is the ratio of liver weight to body weight. The liver and spleen indices of the CTC group were increased significantly by 39.30 and 34.84% compared with the CTL group and by 35.71 and 11.76% compared with the CVSL group, the observation of hepatomegaly in CTC mice indicating that the CCl_4_ injection could successfully cause liver injury and spleen enlargement in mice. Compared with the CTC group, the liver index in the silymarin and CVS-treated hepatic injury groups decreased by 14.11% (CTC.CVSH), 13.27% (CTC.CVSL), and 13.13% (CTC.SIL) (Figure 3A), indicating that the preventive feeding of CVS (both high- and low-dose) and silymarin could significantly reduce the impact of CCl_4_ on the liver index. Compared with the CTC group, the spleen indexes in the CTC.CVSL, CTC.CV, and CTC.SIL groups decreased by 28.95, 15.79, and 23.68%, respectively (Figure 3B), indicating that the preventive feeding of low-dose CVS, vinegar supernatant, and silymarin could significantly raise the abnormal spleen index reduction caused by CCl_4_ in mice. Meanwhile, the preventive feeding of high-dose CVS could only improve the abnormal spleen index to a certain extent.

Liver tissue in the CTL group appeared reddish-brown and shiny with normal lobular architecture, central veins, and radiating hepatic cords (Figure 3C). Meanwhile, in the CTC group, most of the normal hepatic lobules were destroyed or had disappeared. Additionally, the nucleus in the CTC group was squeezed away from the center. The CTC.CSVL and CTC.SIL groups demonstrated a preventive effect in the arrangement of hepatic lobules and nucleus location (Figure 3C).

### CVS Pre-treatment Lowered Mice Serum ALT and AST Levels After CCl_4_ Administration

Serum ALT and AST contents in the CTC group were 10.68 times and 8.96 times higher than that in the CTL group, respectively (Figures 3D,E), proving that CCl_4_ injection could cause liver injury in mice and reduce serum transaminase levels. Compared with the CTC group, the serum ALT and AST levels in the CTC.CVSH, CTC.CVSL, and CTC.SIL groups decreased significantly. This result indicated that the preventive feeding of CVS (both high- and low-dose) or silymarin could significantly restrain the severe increase of serum ALT and AST levels caused by the CCl_4_ injection in mice.

### CVS Pre-treatment Minimized Hepatic MDA, SOD, and CAT Levels Changing in CCl_4_-Induced Liver Injury Mice

As shown in Figure 3F, compared with the CTL group, the MDA content in liver tissue of the CTC group increased by 73.02%. The change of MDA content indicated that the CCl_4_ injection promoted the lipid peroxidation process leading to the accumulation of MDA in the liver. Compared with the CTC group, the hepatic MDA content in the CTC.CVSH, CTC.CVSL, and CTC.SIL groups decreased by 31.19, 28.44, and 41.28%, respectively. It could be possible that the oxidative damage of hepatocytes could be significantly prevented by feeding CVS (both high- and low-dose) and silymarin in advance.

Superoxide dismutase is one of the important antioxidant enzymes that can scavenge free radicals in the body. The CTC group has a significantly lower level of SOD compared with the CTL, CTC.CVSL, CTC.CV, and CTC.SIL groups, whereas there was no statistical difference between the CTL group and the CVSL, CTC.CVSH, CTC.CVSL, CTC.CV, and CTC.SIL groups.

As shown in Figure 3G, compared with CTL, the hepatic SOD activity of CTC mice decreased by 48.74%, indicating that CCl_4_ significantly reduced the activity of SOD, thereby reducing the ability to eliminate free radicals in liver tissue. Compared with CTC, the hepatic SOD activity of CTC.CVSL, CTC.CV, and CTC.SIL mice increased by 104.97, 124.37, and 112.88%, respectively. This result indicated that the feeding of low-dose CVS, vinegar supernatant and silymarin could significantly restrain the hepatic SOD inactivation caused by the CCl_4_ injection.

Compared with CTL, the hepatic CAT activity of CTC mice decreased by 36.25% (Figure 3H), which indicates that the CCl_4_ injection reduces the CAT activity and, thus, reduces the ability of the liver to decompose hydroxyl radicals. Compared with CTC, the hepatic CAT activity of CTC.CVSL and CTC.SIL mice increased by 58.77 and 72.95%, respectively, indicating that feeding low-dose CVS and silymarin in advance could restrain the hepatic CAT inactivation resulting from CCl_4_ and protect the stable internal environment of cells.

### CVS Influenced Gut Microbiota in Mice

An average of 75,536 valid reads for 28 samples were obtained, and the effective rate of quality control was 94.76%. A total of 606 OTUs were obtained from 16s rRNA sequencing, in which 250 OTUs were annotated to genus level and 60 OTUs were annotated to species level.

The rarefaction curve of the 16s rRNA sequencing result was smooth and flat (Supplementary Figure 2A), and the Good's coverage index was >99.8% (Supplementary Figure 2B). Therefore, the sequencing depth of this study is sufficient to study the majority of bacteria in the intestinal tract with high reliability. Compared with the CTL group, Chao 1, and ACE in CTC decreased significantly. On the contrary, CVS (both high- and low-dose) and vinegar supernatant could decrease the Chao1 index and ACE index in mice with liver injury (Supplementary Figures 2C,D). Although only low-dose CVS could significantly decrease the Chao1 index, it is certain that vinegar sediment and vinegar supernatant could reverse the richness of intestinal microorganisms in mice with liver injury. In addition, there was no clear difference between the Shannon and Simpson indices of gut microorganisms among different groups (Supplementary Figures 2E,F).

**Figure 4:**
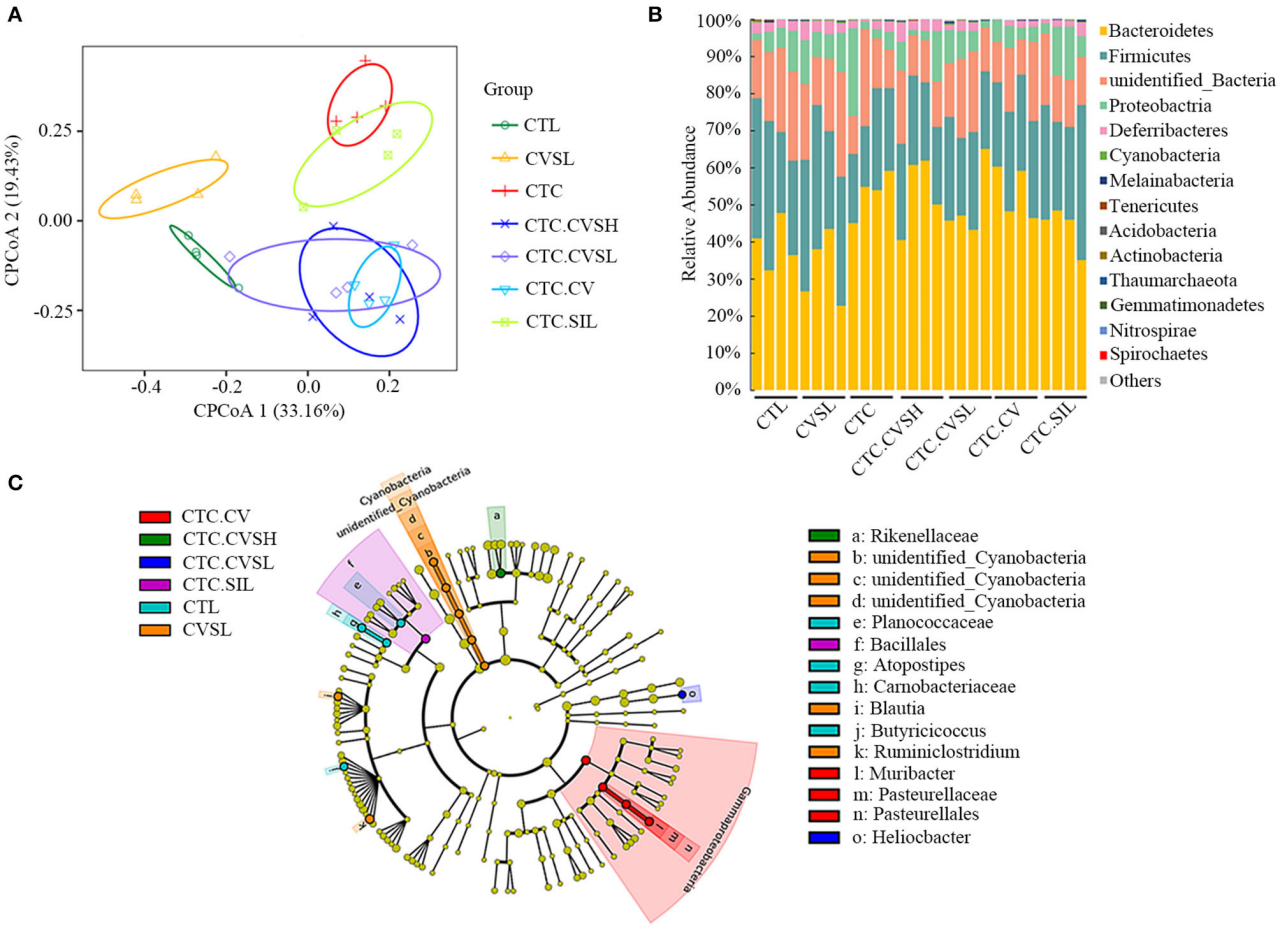
16s rRNA sequencing result of mice gut microbiota in different groups. **(A)** Constrained principal coordinate analysis (CPCoA) according to Bray–Curtis distance matrix was carried out to access beta diversity among the CTL group (green hollow circle), the CVSL group (yellow hollow triangle), CTC group (red line), the CTC.CVSH group (blue cross), the CTC.CVSL group (purple hollow diamond), the CTC.CV group (sky blue hollow inverted triangle), and the CTC.SIL group (light green hollow square); **(B)** bar plots showing the relative abundance and distribution of the phyla in different groups; **(C)** differential bacterial composition of the different groups based on linear discriminant analysis effect size (LEfSe) analysis. Cladogram showing different abundant taxa between samples from different groups (LDA scores > 1.5, *p* < 0.1), p, phylum; c, class; o, order; f, family; g, genus.

Constrained principal coordinate analysis (CPCoA, Figure 4A) based on the Bray–Curtis distance matrix revealed that samples in the same treatment group could be well-clustered. The CVSL and CTL groups could separate from other groups in the CPCoA1 axis with 33.16% representative, whereas the CTC group could separate from the CTC.CVSH, CTC.CVSL, and CTC.CV groups in CPCoA2 axis with 19.43% representative. The CTC.CVSH, CTC.CVSL, and CTC.CV groups showed some overlap among individuals and were clustered together. This result indicated that the CCl_4_ intervention changed the gut microbiota structure of mice.

At the phylum level (Figure 4B), *Bacteroidetes, Firmicutes*, and *Proteobacteria* were the dominant phyla in the gut microbial communities in all samples. At the genus level (Supplementary Table 3), *Helicobacter, unidentified*_*Muribaculaceae, Bacteroides, Alistipes, unidentified_Ruminococcaceae, Ruminiclostridium, Alloprevotella, unidentified_Desulfovibrionaceae*, and *unidentified_ Rhodospirillales* were detected in all samples; *unidentified_Muribaculaceae, Helicobacter, Alloprevotella*, and *Bacteroides* were the dominant genera in the gut microbial communities in all samples. According to the LEfSe analyses (Figure 4C, Supplementary Figure 3), we observed that the gut microbial community selected by the latent Dirichlet allocation (LDA) algorithm of three comparisons, CTC.CVSL vs. CTC, CTC.CVSH vs. CTC, and CTC.SIL vs. CTC had *Alphaproteobacteria* phylum in common.

Compared with the CTL group, the relative abundance of three genera (*unidentified_Clostridiales, Lactobacillus*, and *Anaerovorax*) in CTC mice increased significantly, whereas the relative abundance of ten genera (*Ruminiclostridium, Anaerotruncus, unidentified_Ruminococcaceae, Desulfovibrio, Atopostipes, Peptococcus, Sporosarcina, Blautia, Catabacter*, and *Jeotgalicoccus*) decreased significantly (Supplementary Figure 4). High-dose CVS could reverse the relative abundance of *Peptococcus, Lactobacillus*, and *Catabacter* of mice with liver injury. The relative abundance of *Peptococcus* is positively correlated with butyric acid (35). The relative abundance of *Parabacteroides, Sporosarcina, Staphylococcus, Paraprevotella*, and *Erysipelatoclostridium* in healthy mice treated with low-dose CVS was significantly decreased, and the relative abundance of *Stenotrophomonas, Candidatus*, and *Soleaferrea* was significantly increased (Supplementary Figure 5).

### CVS Pre-treatment Reversed Part of Gut Microbiota Changed by CCl_4_ Treatment

**Figure 5:**
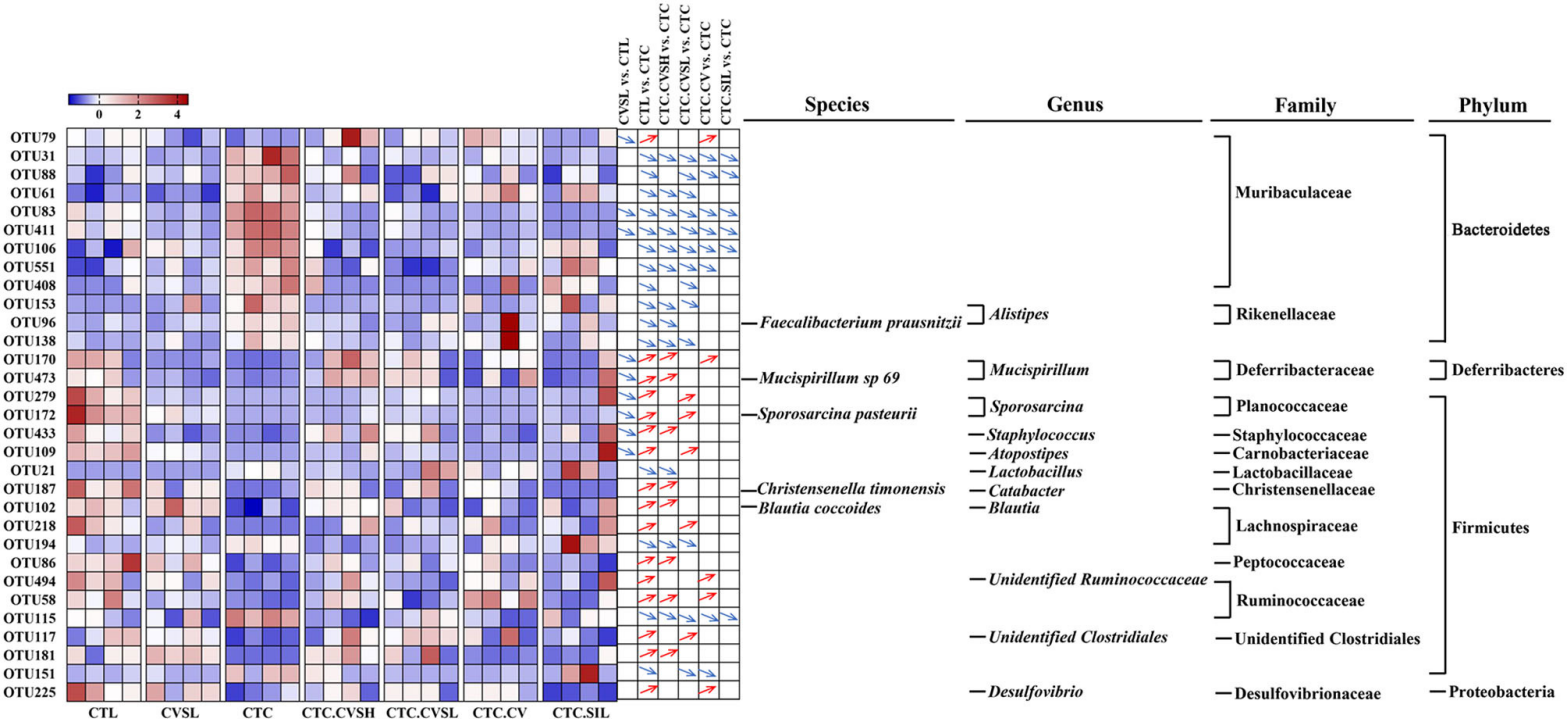
Changes of 31 core operational taxonomic units (OTUs) among different groups. Heatmap showing the relative abundance of 31 core OTUs in each sample among different groups. Red squares represent relatively high abundances, and blue squares represent relatively low abundances. The differentiation and significance of OTU abundances between groups are shown on the right side of the heatmap. For each comparison, a blue down arrow represents the OTU decreased significantly in the front group, and a red up arrow represents the OTU increased significantly in the front group. The taxonomy of 31 core OTUs was annotated on the right side of comparisons. The significances were calculated with Kruskal–Wallis test, and *p* ≤ 0.05 was considered significant.

Compared with the CTL group, 69 OTUs (27 increased, 42 decreased) were significantly altered in the CTC group. Compared with the CTC group, 31 OTUs were significantly altered in total in the CTC.CVSH, CTC.CVSL, CTC.CV, and CTC.SIL groups. These 31 OTUs were then further analyzed (Figure 5) as interesting OTUs.

Among the 31 OTUs altered by CCl_4_, 28 OTUs were reversed by high-dose CVS or low-dose CVS, and 10 OTUs were reversed by both high-dose CVS and low-dose CVS. Among these, only OTU153 was annotated at the genus level, namely *Alistipes*. Sulfobacin B, a lipid consisting of 18 different fatty acid chains, was found to increase in mice that were fed with a high-fat diet, only produced by *Alistipes* and *Odoribacter* (36). In addition to these 10 OTUs, only 4 OTUs reversed by high-dose CVS were identified at the species level, namely OTU96 (*Faecalibacterium prausnitzii*), OTU473 (*Mucispirillum* sp. 69), OTU187 (*Christensenella timonensis*), and OTU102 (*Blautia coccoides*), and only 3 OTUs were identified at the genus level, including OTU170 (*Mucispirillum*), OTU433 (*Staphylococcus*), and OTU21 (*Lactobacillus*).

### Predicted Metabolic Functions of Gut Microbiota

**Figure 6:**
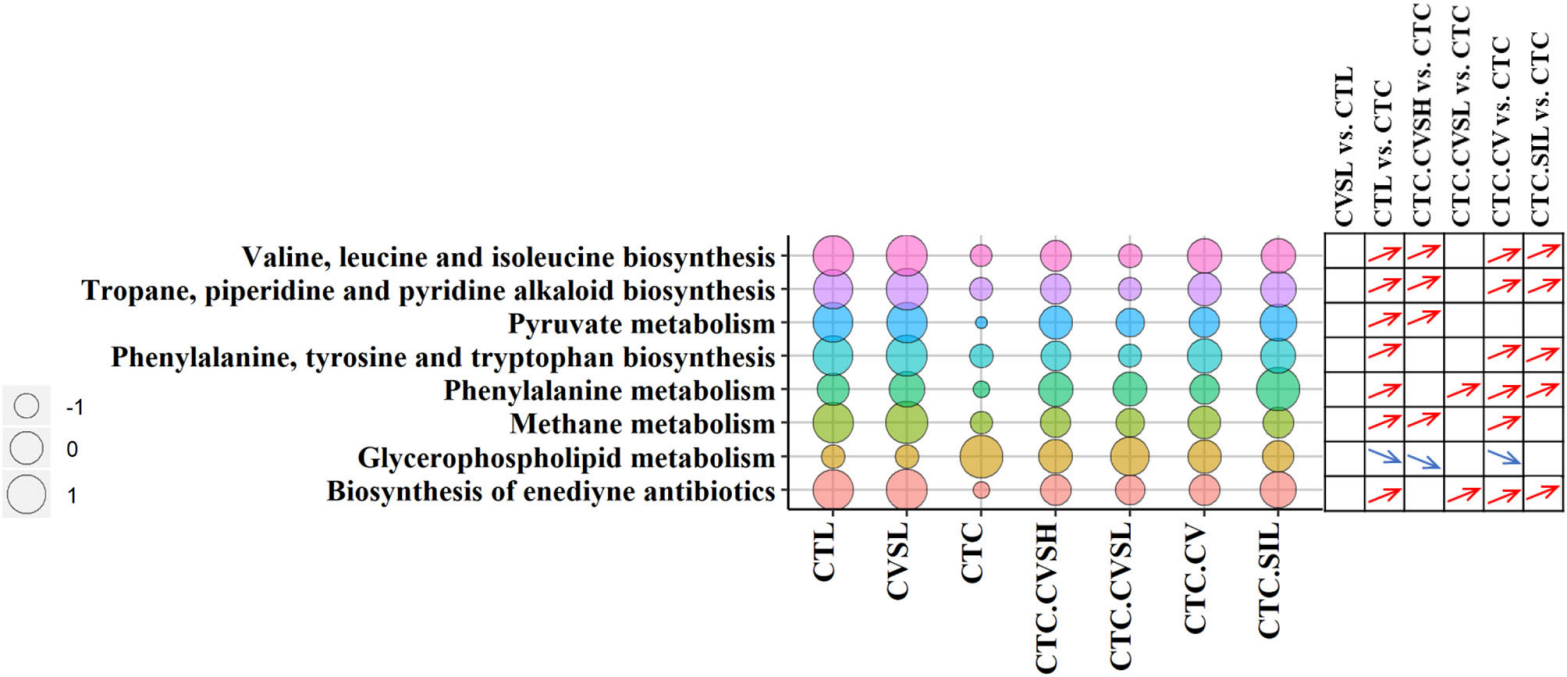
Pathway enrichment in different groups. The bubble chart represents the pathway enrichment predicted by Tax4Fun (37) software among different groups. The size of circulars represents the operational taxonomic unit (OTU) enrichment of pathways, and the different bubble colors represent the different pathways. The differentiation and significance of pathway enrichment between groups are shown on the right side of the bubble chart. For each comparison, a blue down arrow represents the pathway enriched significantly low in the front group, and a red up arrow represents the pathway enriched significantly high in the front group. The significances were calculated with Kruskal–Wallis test, and *p* ≤ 0.05 was considered significant.

Function prediction analysis of Tax4Fun (37) was implemented. Compared with the CTL group, the relative abundance of genes predicted in 36 metabolic pathways were significantly altered in the CTC group, among which glycerophospholipid metabolism, methane metabolism, tropane, piperidine and pyridine alkaloid biosynthesis, valine, leucine and isoleucine biosynthesis, and pyruvate metabolism were reversed by high-dose CVS (Figure 6).

The relative abundance of pentose and glucuronate interconversions was significantly reduced in normal mice fed with low-dose CVS, which were increased by CCl_4_ and decreased by prevention of CVS (both low-dose and high-dose) in advance, but there was no statistical difference between CVSL and CTC.CVSL groups (Supplementary Figure 6).

### Correlations Between Mice Microbiota Structures and Physicochemical Indexes

**Figure 7:**
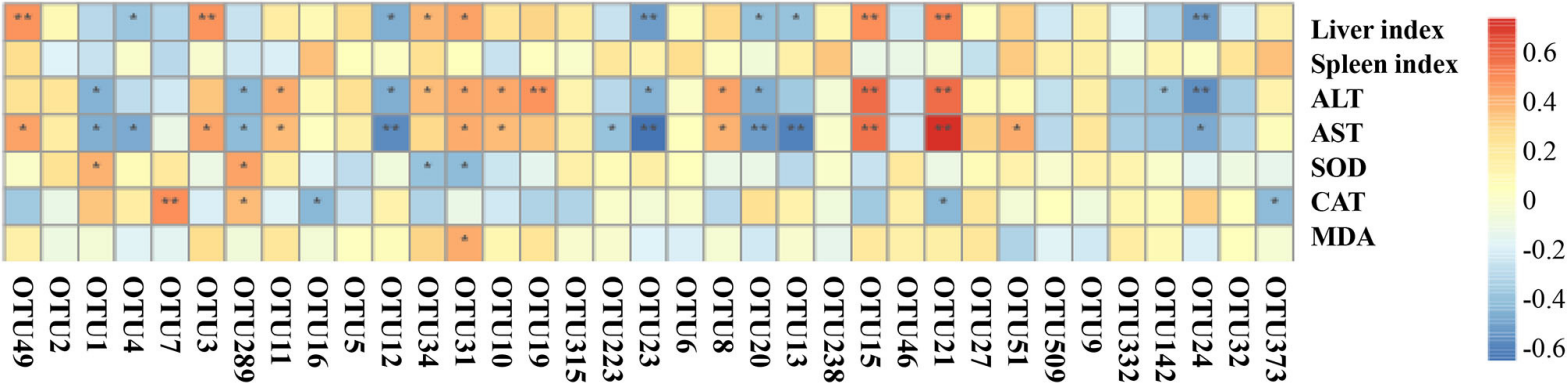
The correlation between operational taxonomic units (OTUs) and physicochemical indices. Heatmap showing the correlations between OTUs and physicochemical indices, the correlations were calculated with Spearman's rank correlation coefficient. Warm colors represent the positive correlation between the OTU and the index, cold colors represent the negative correlation between the OTU and the index. The significances were calculated from Spearman's rank correlation coefficient. The stars represent the significance of correlations: **p < 0.01, *p < 0.05.

The degree of liver injury is positively correlated with liver and spleen indices, and ALT, AST, and MDA levels. However, it is negatively correlated with CAT and SOD activities. Four OTUs, namely OTU5 (*Bacteroides*), OTU34 (*Alistipes*), OTU31 (Muribaculaceae), and OTU19 (*Bacteroides sartorii*), were positively correlated with liver and spleen indices and ALT, AST, and MDA levels. The same OTUs were also negatively correlated with CAT and SOD activities (Figure 7). It has been reported that the severity of NAFLD is positively correlated with Bacteroides abundance (38). OTU1 (*Helicobacter ganmani*), OTU7 (*Rhodospirillales*), and OTU289 (*Helicobacter ganmani*) were negatively correlated with liver and spleen indices and ALT, AST, and MDA levels, whereas the same OTUs were positively correlated with CAT and SOD activities.

As shown in Supplementary Figure 7, low-dose CVS could significantly reduce the relative abundance of OTU34 (*Alistipes*), and CVS (both high- and low-dose), vinegar supernatant, and silymarin could significantly reduce the relative abundance of OTU31 (Muribaculaceae). It was speculated that the preventive effect of low-dose CVS on liver injury might be related to OTU34 (*Alistipes*) and OTU31 (Muribaculaceae), and the preventive effects of high-dose CVS, vinegar supernatant, and silymarin on liver injury may be related to OTU31 (Muribaculaceae). So, the degree of liver injury might be positively correlated with the relative abundance of OTU34 (*Alistipes*) and OTU31 (Muribaculaceae).

## Discussion

Traditionally, CSV was made during the cereal vinegar aging process for years. As an insoluble mixture, a typical understanding of the formation of vinegar sediment is because of polymerizations, gravity, (bio-)chemistry reactions and evaporation of undegraded starch, protein, pectin, and cellulose (39). Our untargeted metabolomics analysis of CVS proved this understanding, *p*-coumaroylagmatine was likely carried from barley (40). It is reported that *Bacillus subtilis* was isolated from rice vinegar sediments, and the formation of Shanxi aromatic vinegar sediment crystals was caused by supersaturation of calcium oxalate (41). In our study, there was a decoction step before the aging process in CVS production, which was aimed to kill living bacteria. Our qPCR result with amplifier 505F and 806R in CVS was negative, neither was Raman spectroscopic result, so there were no bacteria existed in the CVS sample.

It is reported that total polyphenols and total flavonoids were increasing during the Shanxi aged vinegar aging process (39). CVS total polyphenol and total flavonoid contents were relatively higher than Shanxi aged vinegar (42, 43). Qiu et al. (24) reported that oat vinegar showed a strong antioxidant activity than rice vinegar because oat vinegar had higher amounts of polyphenols. Nie et al. (44) demonstrated that the polyphenolic extract from apples, particularly from peels, can be explored as a chemopreventive or chemotherapeutic agent against oxidative-stress-related liver disorders. Flavonoids have been recognized as hepatoprotective compounds. Wang et al. (45) reported that flavonoids extracted from Iris plants showed inhibitory effects on CCl_4_-induced rat liver fibrosis. Hu et al. (46) found that purified tartary buckwheat flavonoid fractions, containing 53.6% rutin and 37.2% quercetin, could prevent trimethylamine N-oxide (TMAO)-induced vascular dysfunction and hepatic injury.

The CVS pretreatment could statistically reduce the MDA increasing, SOD decreasing, and CAT decreasing caused by CCl_4_ injection. MDA was used as a marker of oxidative stress-induced liver injury. SOD and CAT activities were used to evaluate the changes in the antioxidative system in liver tissues (47). Our results suggested that CVS pretreatment could enhance the efficiency of scavenging free radicals and decomposing hydroxyl radicals in the liver.

Interestingly, as shown in Supplementary Figure 3, the different features between the CTC and CTL groups, the CTC and CTC.CVSL groups, the CTC and CVSH groups, and the CTC vs. CTC.SIL groups had common ground. Alphaproteobacteria were relatively low in the CTC group, which indicated the liver injury may affect the gut Alphaproteobacteria (48). *Staphylococcus lentus* had the highest LDA score between the CTC and CTC.SIL groups, however, was not significant in neither the CTL vs. CTC, the CTC vs. CTC.CVSL nor the CTC vs. CTC.CVSH comparisons. This result indicated *Staphylococcus lentus* may respond particularly under silymarin-pretreated acute liver injury mice. Bacteroidia had significant differentiation between CTC and CTL, CTC and CTC.CVSL, and CTC and CTC.CVSH groups but had no significance between CTC and CTC.SIL groups. This result indicated the different regulations to gut microbiota of CVS and silymarin.

*Alistipes* is a genus that may have protective effects against some diseases, including liver fibrosis, colitis, cancer immunotherapy, and cardiovascular disease (49). According to our result, low-dose CVS intake can significantly increase the relative abundance of *Alistipes*, with CCl_4_ administration significantly decreasing the relative abundance of *Alistipes*. Muribaculaceae family belongs to the Bacteroides class, contributes to propionate production, and may have a correlation with longevity enhancement (50). In our study, Muribaculaceae family OTUs significantly increased after CCl_4_ administration without pretreatments. However, Muribaculaceae abundances could shift from increasing to relatively stable under either low- and high-dose CVS, or silymarin feeding. Moreover, OTU34 (*Alistipes*) and OTU31 (Muribaculaceae) showed positive correlations with liver ALT, AST levels, and a negative correlation with liver SOD levels. Although there was no direct evidence of CVS had a hepatoprotective effect through the gut–liver axis, OTU34 (*Alistipes*) and OTU31 (Muribaculaceae) were interesting findings that may play important roles in CVS intake response and hepatoprotective effects.

It has been reported that the significant increase of *Lactobacillus* is related to hepatopathy (51) and colitis (52). It is speculated that the hepatoprotective effect of high-dose CVS might be related to the relative abundance of *Lactobacillus, Anaerotruncus*, and *Peptococcus*. By contrast, low-dose CVS could only reverse the relative abundance of *Atopostipes* and *Sporosarcina* of mice with liver injury. *Atopostipes* is a gram-positive bacterium that metabolizes valine and tryptophan into short-chain fatty acids and indole and is positively correlated with phenol, indole, isobutyric acid, and isovaleric acid (53). Perhaps low-dose CVS could enhance the metabolism of valine and tryptophan in the intestine. Vinegar supernatant could significantly reverse the relative abundance of *Desulfovibrio* and *Catabacter* of mice with liver injury, and notably, the latter reversed by high-dose CVS. Silymarin could only reverse the relative abundance of *Jeotgalicoccus* of mice with liver injury.

Cereal vinegar sediment may have some effect on the intestinal mucosal barrier. Among the OTUs affected by low-dose CVS but not altered by high-dose CVS, only 1 OTU and 3 OTUs were identified at the species level and genus level, respectively, namely OTU172 (*Sporosarcina pasteurii*), OTU279 (*Sporosarcina*), OTU109 (*Atopostipes*), and OTU117 (*unidentified_Clostridiales*). A total of 36 OTUs (11 increased, 25 decreased) were altered in healthy mice fed with low-dose CVS, 2 OTUs of which were increased by CCl_4_ and decreased after the intervention of CVS (both high- and low-dose), vinegar supernatant, and silymarin. The relative abundance of the 2 OTUs annotated with the Muribaculaceae family in healthy mice treated with low-dose CVS also decreased significantly.

Although the authors measured CVS with multiple methods, there are still several uncertain components inside the CVS like the structure of polysaccharides, the composition of flavonoids, and the composition of insoluble parts. The authors illustrated a potential functional use of CVS, there are several shortages in this study, for example, the number of mice used in 16S rRNA sequencing and the lack of bacteria load measurement. Further studies are needed to clarify the mechanisms of how CVS treatment influences the hepatoprotective effect.

## Data Availability Statement

The datasets presented in this study can be found in online repositories. The names of the repository/repositories and accession number(s) can be found in the article/Supplementary Material.

## Ethics Statement

The animal study was reviewed and approved by the Institutional Animal Care and Use Committee of Jiangnan University, Wuxi, China.

## Author Contributions

QG: contributed to methodology, formal analysis, data curation, writing—review and editing, and visualization. TG: involved in investigation and writing original draft. Z-ML: presented conceptualization, validation, and project administration. YG: involved in supervision and methodology. WD: investigated. Y-LR: provided resources. X-JZ: performed methodology. L-JC: validated. J-SS: supervised. Z-HX: contributed to conceptualization, supervision, and funding acquisition. All authors contributed to the article and approved the submitted version.

## Funding

This study was supported by the National Key R&D Program of China (Grant Nos. 2018YFC1603800 and 2018YFC1603802), the National Natural Science Foundation of China (Grant No. 31771967), and National First-Class Discipline Program of Light Industry Technology and Engineering (Grant No. LITE2018-11).

## Conflict of Interest

The authors declare that the research was conducted in the absence of any commercial or financial relationships that could be construed as a potential conflict of interest.

## Publisher's Note

All claims expressed in this article are solely those of the authors and do not necessarily represent those of their affiliated organizations, or those of the publisher, the editors and the reviewers. Any product that may be evaluated in this article, or claim that may be made by its manufacturer, is not guaranteed or endorsed by the publisher.

## Supplementary Material

Supplementary Figure 1 Weight change rate of cereal vinegar sediment (CVS) in liver injury mice. Plot showing the body weight changing in different groups. The different colors represent different groups, and error bars represent SD.

Supplementary Figure 2 Alpha diversity of gut microbiota. **(A)** Rarefaction curves; Alpha diversity indices, including Good's coverage **(B)**, Chao1 **(C)**, ACE **(D)**, Shannon **(E)**, and Simpson **(F)**. Different letters represent a significant difference between groups calculated with the Kruskal–Wallis one-way ANOVA, and error bars represent SD.

Supplementary Figure 3 Linear discriminant analysis effect size (LEfSe) results based on operational taxonomic unit (OTU) level in different comparisons. **(A)** CTC vs. CTL. Green bars represent OTUs were relatively higher in the CTL group, red bars represent OTUs were relatively higher in the CTC group; **(B)** CTC vs. CTC.SIL. Green bars represent OTUs were relatively higher in the CTC.SIL group, red bars represent OTUs were relatively higher in the CTC group; **(C)** CTC vs. CTC.CVSL. Green bars represent OTUs were relatively higher in the CTC.CVSL group; red bars represent OTUs were relatively higher in the CTC group; **(D)** CTC vs. CTC.CVSH. Green bars represent OTUs were relatively higher in the CTC.CVSH group; red bars represent OTUs were relatively higher in the CTC group.

Supplementary Figure 4 Operational taxonomic units (OTUs) significantly different between CTL and CTC at “genus” level. **(A)** A genus with relative abundance higher than 0.1%; **(B)** genus with relative abundance <0.1%. Different letters represent a significant difference between groups calculated with the Kruskal–Wallis one-way ANOVA, and error bars represent SD.

Supplementary Figure 5 Operational taxonomic units (OTUs) significantly different between CTL and CVSL at “genus” level. Different letters represent a significant difference between groups calculated with the Kruskal–Wallis one-way ANOVA, and error bars represent SD.

Supplementary Figure 6 Relative abundance of operational taxonomic units (OTUs) predicted relating to pentose and glucuronate interconversions pathway. Bar plot showing the relative abundances of selected OTUs in different groups. Different colors represent different groups. Different letters represent a significant difference between groups calculated with the Kruskal–Wallis one-way ANOVA, and error bars represent SD.

Supplementary Figure 7 Relative abundance of some operational taxonomic units (OTUs) related to physicochemical indices. Bar plot showing the relative abundances of selected OTUs in different groups. Different colors represent different groups. Different letters represent a significant difference between groups calculated with the Kruskal–Wallis one-way ANOVA, and error bars represent SD.

Supplementary Table 1 Detailed chemical compositions of CVS. ^*^Essential amino acids.

Supplementary Table 2 Metabolites detected in CVS with LC-MSMS analysis.

Supplementary Table 3 OTU table generated by vsearch with SILVA 132 database. Sample ids represent groups and replicates.
