# Supplementary figures and images for: Hepatoprotective Effect of Cereal Vinegar Sediment in Acute Liver Injury Mice and Its Influence on Gut Microbiota
Source: Front Nutr. 2021 Dec 24;8:798273. doi: 10.3389/fnut.2021.798273 (PMC8740290; doi:10.3389/fnut.2021.798273)

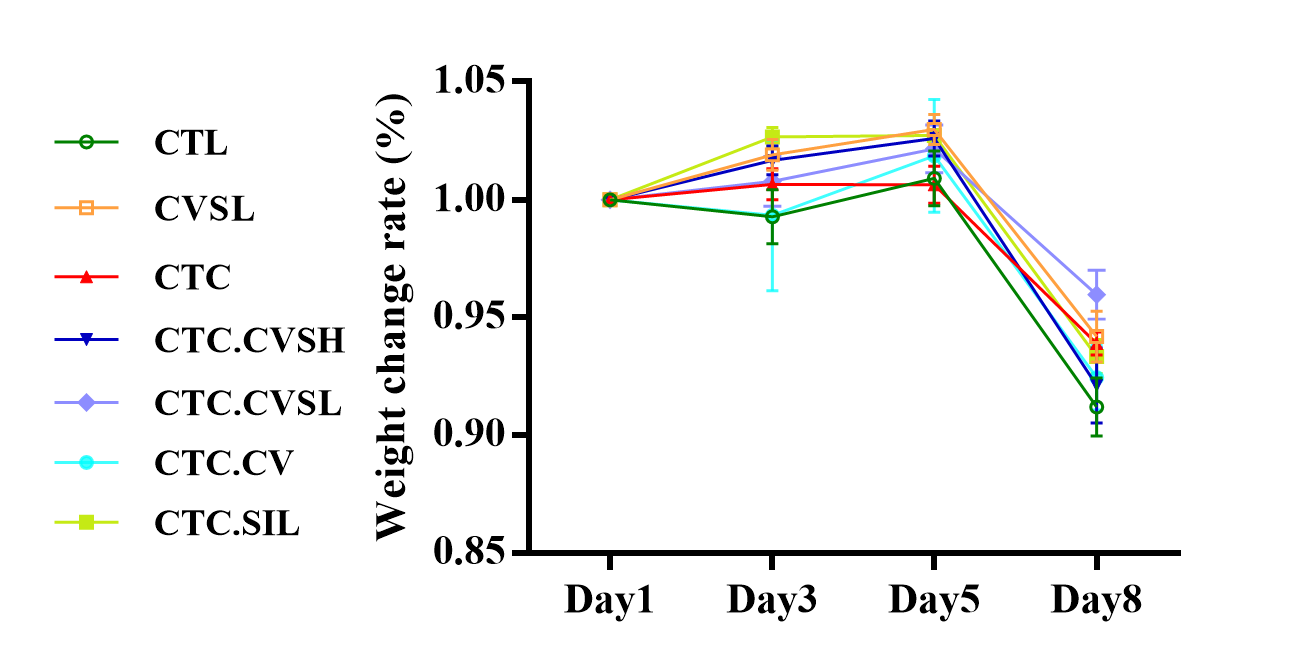

Supplement: Supplementary Figure 1 — Weight change rate of cereal vinegar sediment (CVS) in liver injury mice. Plot showing the body weight changing in different groups. The different colors represent different groups, and error bars represent SD. [file Image_1.TIF]

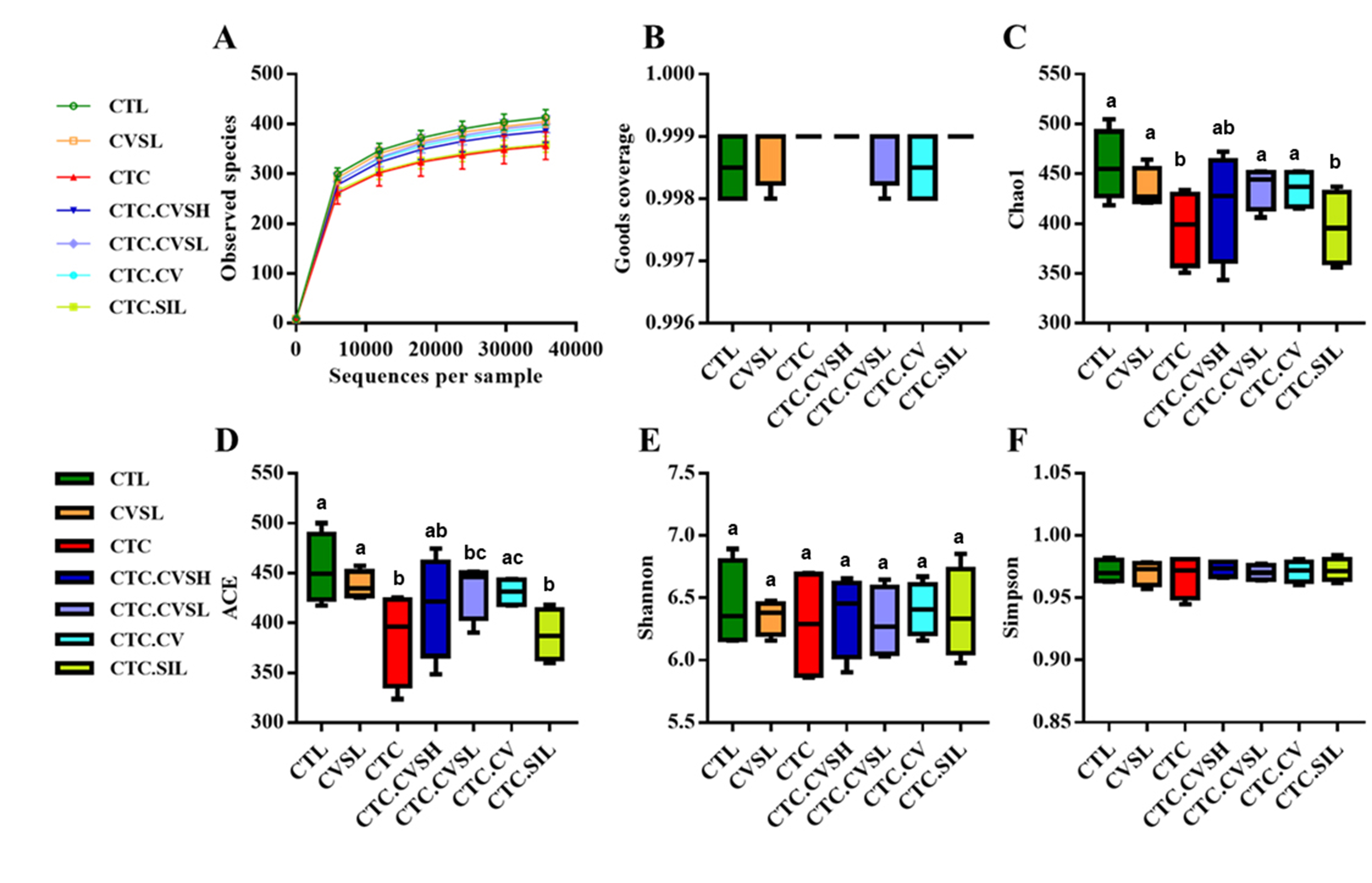

Supplement: Supplementary Figure 2 — Alpha diversity of gut microbiota. (A) Rarefaction curves; Alpha diversity indices, including Good's coverage (B), Chao1 (C), ACE (D), Shannon (E), and Simpson (F). Different letters represent a significant difference between groups calculated with the Kruskal–Wallis one-way ANOVA, and error bars represent SD. [file Image_2.JPEG]

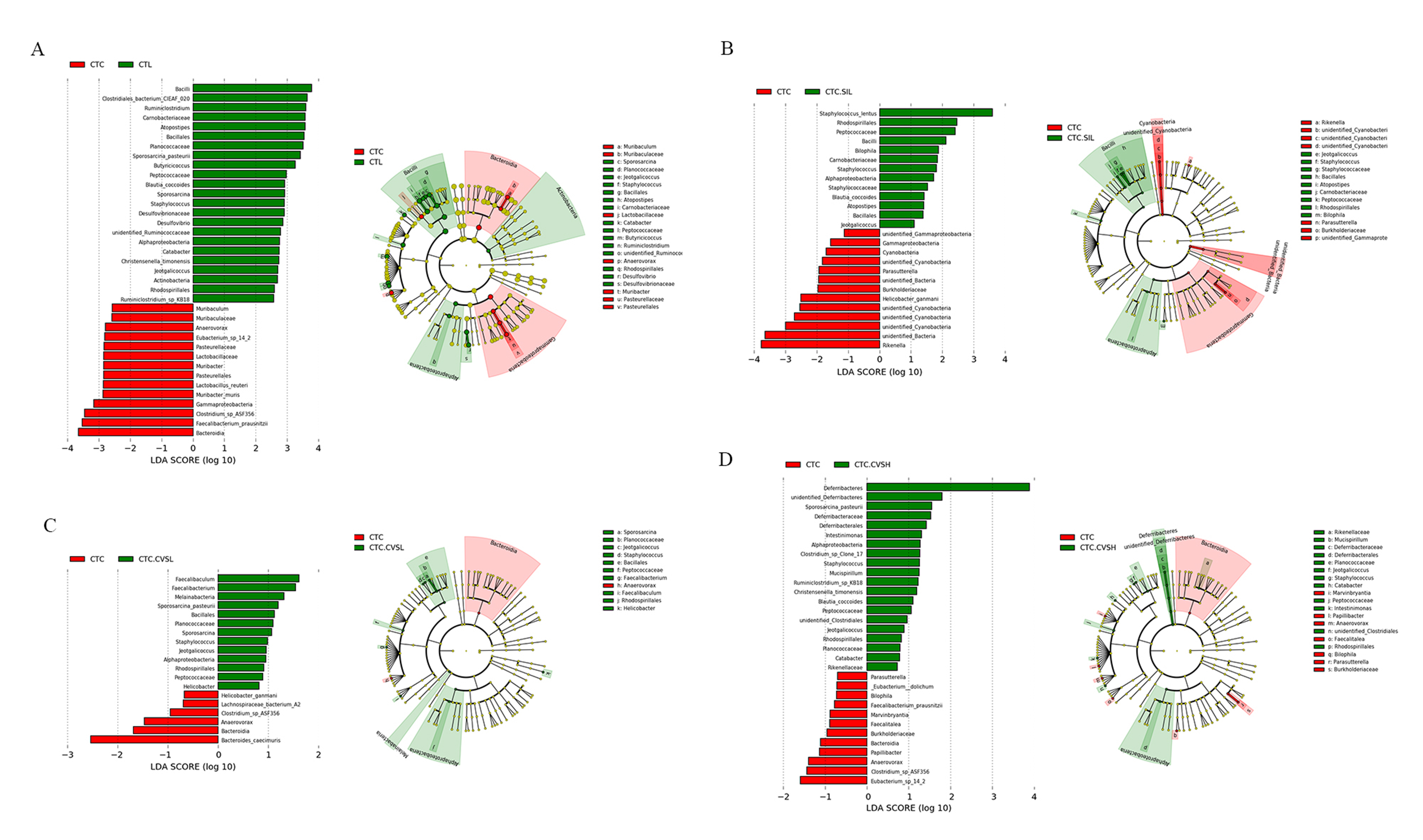

Supplement: Supplementary Figure 3 — Linear discriminant analysis effect size (LEfSe) results based on operational taxonomic unit (OTU) level in different comparisons. (A) CTC vs. CTL. Green bars represent OTUs were relatively higher in the CTL group, red bars represent OTUs were relatively higher in the CTC group; (B) CTC vs. CTC.SIL. Green bars represent OTUs were relatively higher in the CTC.SIL group, red bars represent OTUs were relatively higher in the CTC group; (C) CTC vs. CTC.CVSL. Green bars represent OTUs were relatively higher in the CTC.CVSL group; red bars represent OTUs were relatively higher in the CTC group; (D) CTC vs. CTC.CVSH. Green bars represent OTUs were relatively higher in the CTC.CVSH group; red bars represent OTUs were relatively higher in the CTC group. [file Image_3.JPEG]

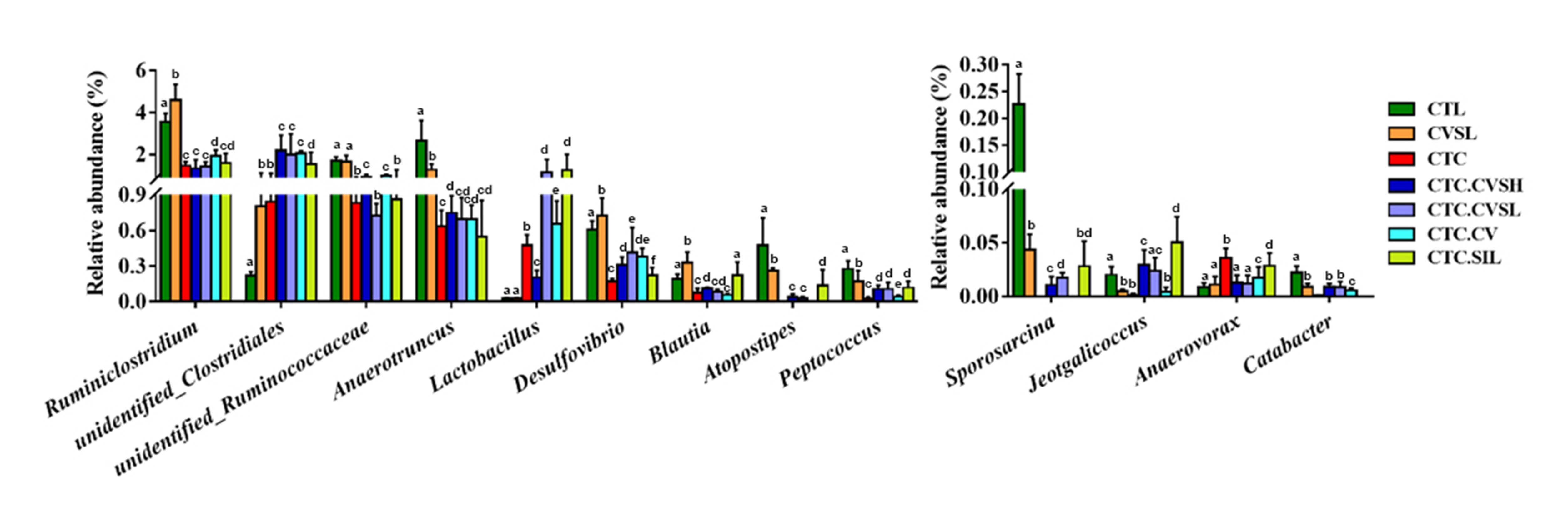

Supplement: Supplementary Figure 4 — Operational taxonomic units (OTUs) significantly different between CTL and CTC at “genus” level. (A) A genus with relative abundance higher than 0.1%; (B) genus with relative abundance <0.1%. Different letters represent a significant difference between groups calculated with the Kruskal–Wallis one-way ANOVA, and error bars represent SD. [file Image_4.JPEG]

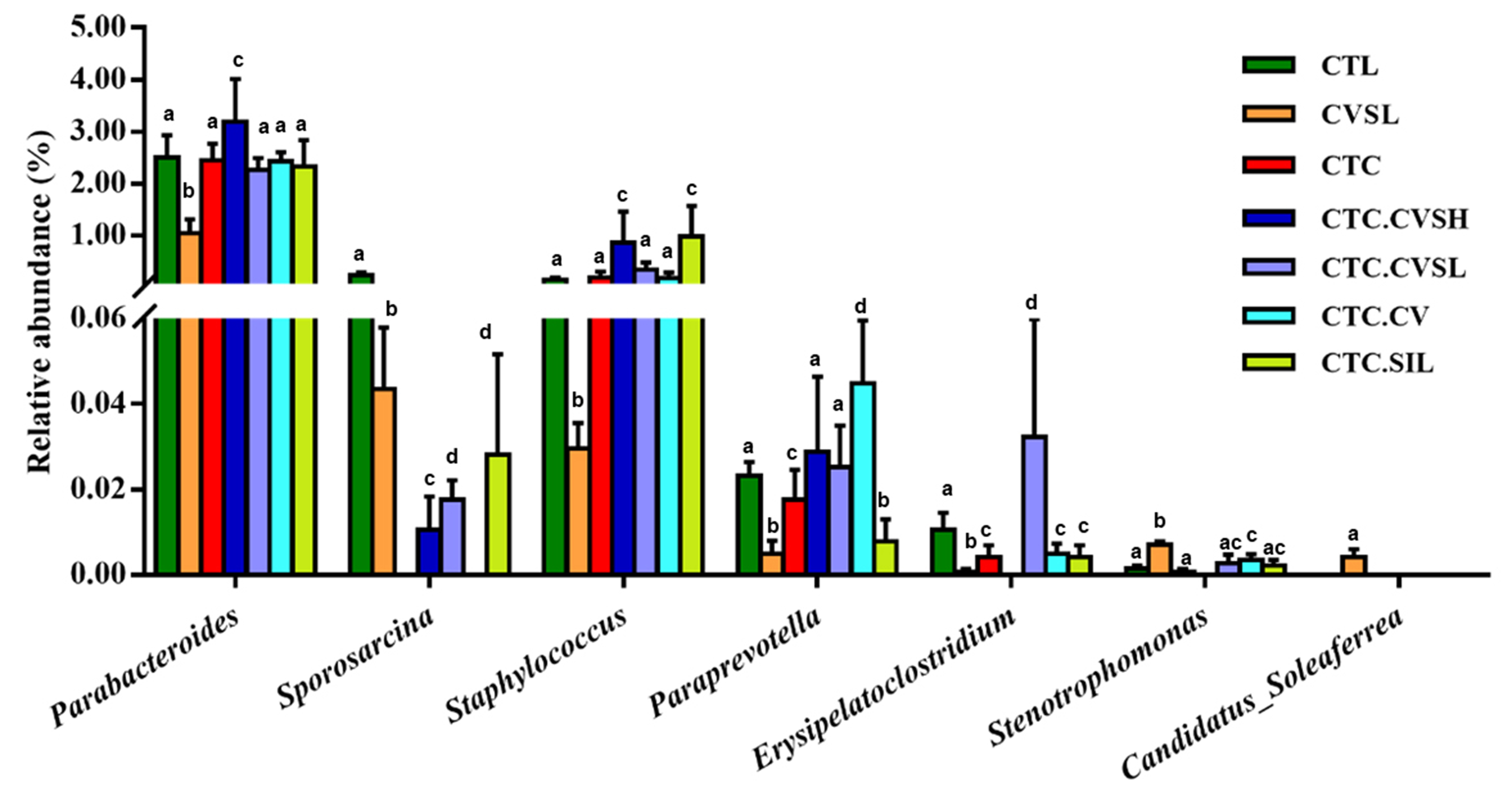

Supplement: Supplementary Figure 5 — Operational taxonomic units (OTUs) significantly different between CTL and CVSL at “genus” level. Different letters represent a significant difference between groups calculated with the Kruskal–Wallis one-way ANOVA, and error bars represent SD. [file Image_5.JPEG]

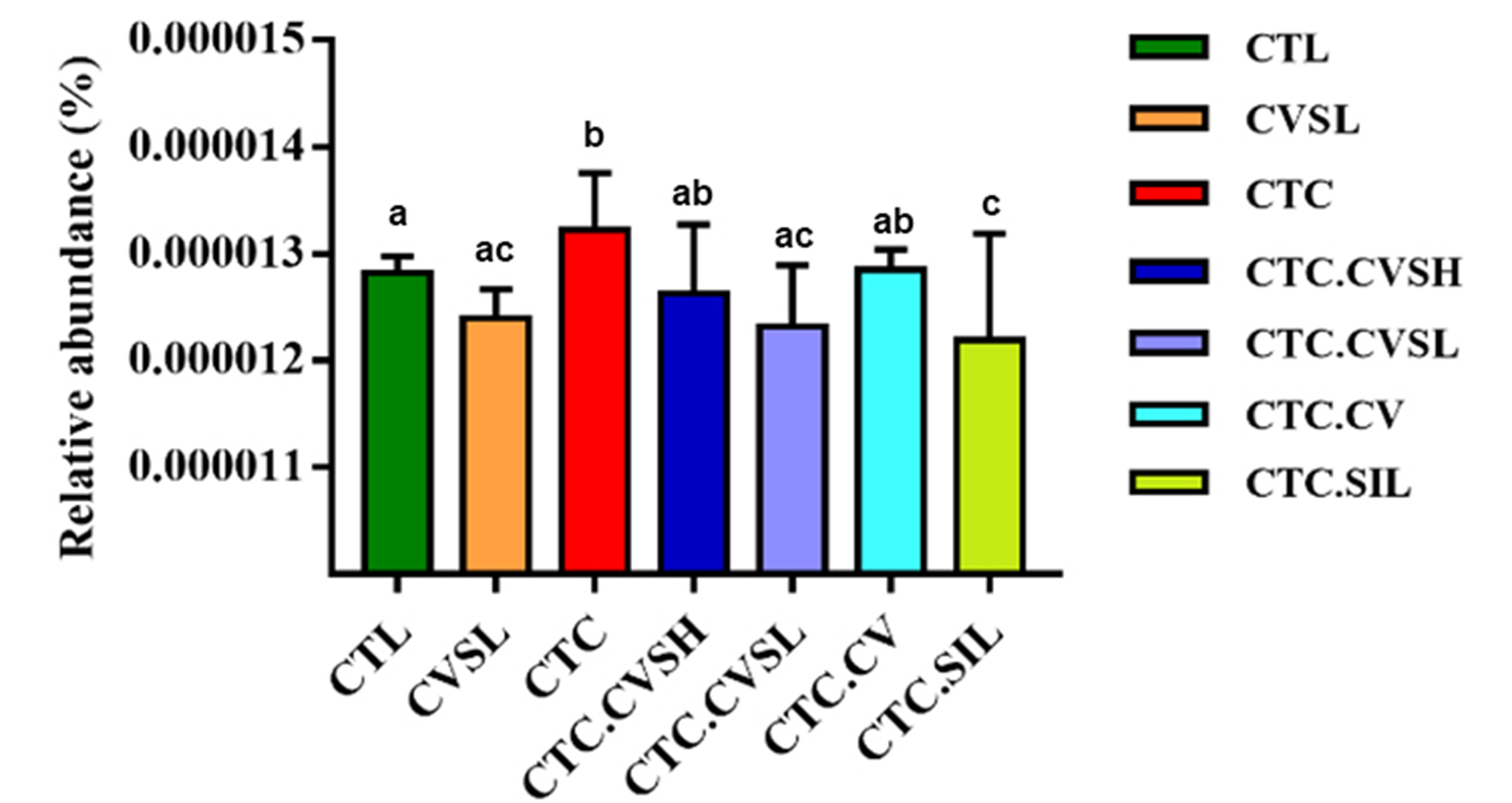

Supplement: Supplementary Figure 6 — Relative abundance of operational taxonomic units (OTUs) predicted relating to pentose and glucuronate interconversions pathway. Bar plot showing the relative abundances of selected OTUs in different groups. Different colors represent different groups. Different letters represent a significant difference between groups calculated with the Kruskal–Wallis one-way ANOVA, and error bars represent SD. [file Image_6.JPEG]

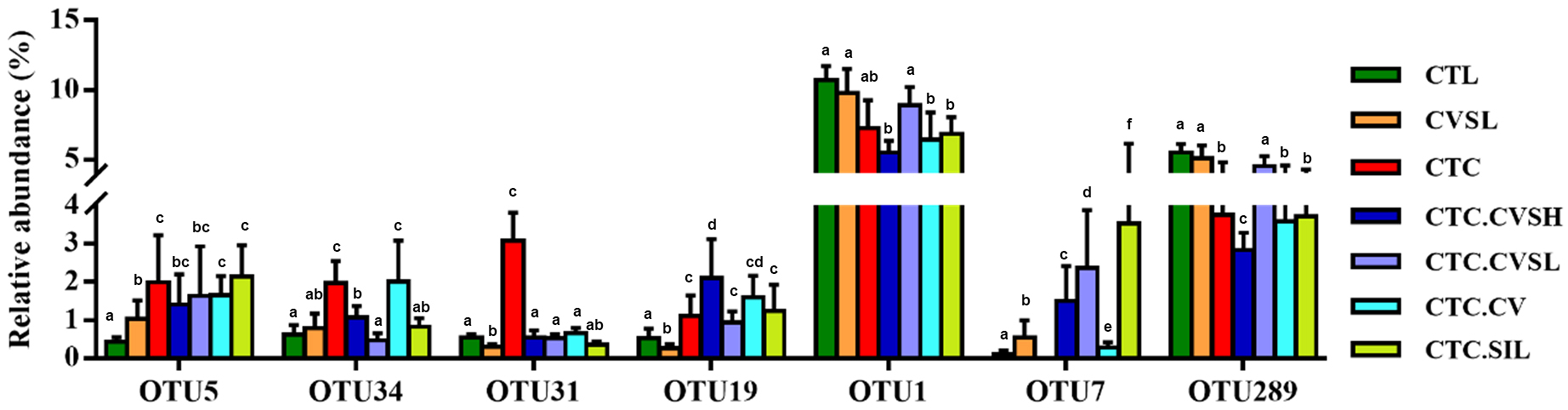

Supplement: Supplementary Figure 7 — Relative abundance of some operational taxonomic units (OTUs) related to physicochemical indices. Bar plot showing the relative abundances of selected OTUs in different groups. Different colors represent different groups. Different letters represent a significant difference between groups calculated with the Kruskal–Wallis one-way ANOVA, and error bars represent SD. [file Image_7.JPEG]
