# Supplementary material for: Hepatoprotective Effect of Cereal Vinegar Sediment in Acute Liver Injury Mice and Its Influence on Gut Microbiota
Source: Front Nutr. 2021 Dec 24;8:798273. doi: 10.3389/fnut.2021.798273 (PMC8740290; doi:10.3389/fnut.2021.798273)
Supplement: Supplementary Table 1 — Detailed chemical compositions of CVS. *Essential amino acids. [file Table_1.DOCX]

**Supplementary Table 1. Chemical compositions of CVS**

| components | content (g/100 g) | components | content (g/100 g) |
| --- | --- | --- | --- |
| **Ash** | **3.75** | **Crude protein** | **7.80** |
| **Crude fat** | **0.23** | **Hydrolyzed amino acids** | **4.68** |
| **Carbohydrates** | **23.23** | Glu | 1.68 |
| **Total polyphenols** | **0.40** | Pro | 0.36 |
| **Total flavonoids** | **0.26** | Asp | 0.36 |
| **Organic acids** | **7.23** | Gly | 0.34 |
| **Total saccharides** | **12.08** | Ala | 0.30 |
| Monosaccharide composition | | Arg | 0.15 |
| Gluc | 3.55 | His | 0.14 |
| Xyl | 0.84 | Ser | 0.12 |
| Ara | 0.68 | Tyr | 0.12 |
| Gal | 0.27 | Cys | 0.05 |
| Man | 0.25 | Val* | 0.27 |
| Glc | 0.08 | Leu* | 0.25 |
| **Polysaccharides** | **0.39** | Ile* | 0.15 |
| **Organic acids** | **7.23** | Phe* | 0.13 |
| Acetic acid | 5.29 | Thr* | 0.12 |
| Citric acid | 1.35 | Lys* | 0.10 |
| Succinic acid | 0.35 | Met* | 0.04 |
| Lactic acid | 0.14 | **Free amino acids** | **0.43** |
| Tartaric acid | 0.09 | Glu | 0.04 |
| Pyruvic acid | 0.01 | Pro | 0.02 |
| Oxalic acid | 0.00 | Asp | 0.08 |
|  |  | Gly | 0.02 |
|  |  | Ala | 0.04 |
|  |  | Arg | 0.08 |
|  |  | His | 0.01 |
|  |  | Ser | 0.01 |
|  |  | Tyr | 0.01 |
|  |  | Cys | 0.00 |
|  |  | Val* | 0.03 |
|  |  | Leu* | 0.04 |
|  |  | Ile* | 0.02 |
|  |  | Phe* | 0.01 |
|  |  | Thr* | 0.01 |
|  |  | Lys* | 0.01 |
|  |  | Met* | 0.00 |
|  |  |  |  |

*: Essential amino acids
